# Age-related trabecular bone loss is associated with a decline in serum Galectin-1 level

**DOI:** 10.1186/s12891-021-04272-y

**Published:** 2021-04-27

**Authors:** Wenting Xu, Cheng Ni, Yuxuan Wang, Guoqing Zheng, Jinshan Zhang, Youjia Xu

**Affiliations:** 1 Department of Orthopaedics, The Second Affiliated Hospital of Soochow University, Suzhou, 215004 Jiangsu China; 2 Department of Orthopaedics, Shanghai Jiangong Hospital, Shanghai, 200083 China

**Keywords:** Galectin-1, Osteoporosis, Trabecular bone, Aging, Bone marrow stromal cells

## Abstract

**Background:**

Senile osteoporosis with age-related bone loss is diagnosed depending on radiographic changes of bone and bone mineral density (BMD) measurement. However, radiographic alterations are usually signs of medium-late stage osteoporosis. Therefore, biomarkers have been proposed as indicators of bone loss. In the current study, Galectin-1 (Gal-1) showed age-related decline in mice serum. The role of Gal-1 in osteoporosis has not been investigated so far. Hence, the current study illustrated the relationship of serum Gal-1 level with bone loss.

**Methods:**

We employed 6- and 18-month-old mice to establish an animal model of age-related trabecular bone loss, whose bone density and microstructure were investigated by micro-CT. ELISA was used to measure the levels of Gal-1 in serum. The correlation analysis was performed to illustrate the relationship between serum Gal-1 levels and trabecular bone loss. In addition, immunohistochemistry was used to investigate the abundance of Gal-1 in bone marrow of mice. ELISA and western blot were performed to measure the secretion ability and protein expression of Gal-1 in bone marrow stromal cells (BMSC), hematopoietic stem cells (HSC) and myeloid progenitor (MP) respectively. Flow cytometry was used to measure BMSC number in bone marrow. Finally, male volunteers with age-related BMD decrease were recruited and the relationship between serum Gal-1 and BMD was analyzed.

**Results:**

Gal-1 showed age-related decline in mice serum. Serum Gal-1 was positively associated with BV/TV of femur, tibia and L1 vertebrae in mice. BMSC secreted more Gal-1 compared with HSC and MP. BMSC number in bone marrow was significantly lower in aged mice compared with young mice. Significant attenuation of Gal-1 protein expression was observed in BMSC and HSC from aged mice compared with young mice. Further, we found a decline in serum Gal-1 levels in men with age-related BMD decrease. There was positive correlation between BMD and serum Gal-1 levels in these men.

**Conclusions:**

Age-related trabecular bone loss is associated with a decline in serum Gal-1 level in mice and men. Our study suggested Gal-1 had great potential to be a biomarker for discovering BMSC senescence, diagnosing early osteoporosis and monitoring trabecular bone loss.

**Supplementary Information:**

The online version contains supplementary material available at 10.1186/s12891-021-04272-y.

## Introduction

Bone loss in elderly people, often named as senile osteoporosis, takes place with aging, which leads to bone fragility and increased fractures risk. It has been one of the most severe diseases affecting the elderly population worldwide [1]. The pathophysiological mechanisms underlying senile osteoporosis are currently hot topics with the aim of developing new approaches to diagnose, prevent and treat osteoporotic bone loss in elderly people [1].

The maintenance of bone homeostasis requires a perfect balance between the destructive activity of osteoclasts and the reparative function of osteoblasts which is called “bone remodeling”. The coupling between these processes presupposes an intimate form of cross-talk between the cells and the existence of autocrine/paracrine mechanisms of interaction [2]. When the amount of bone removed by the osteoclasts is generally equal to the amount of bone formed by the osteoblasts, the bone mass could remain stable relatively. However, bone homeostasis is disrupted during aging. Increased bone resorption and/or decreased bone formation can cause bone loss. Several histomorphometric studies on iliac crest biopsies revealed that a decrease in bone formation but not an increase in bone resorption seems to be the principle pathological cause responsible for senile osteoporosis [3].

Bone formation depends on the amount and activity of osteoblasts during bone remodeling. Osteoblasts are differentiated from stem cells present in the non-hematopoietic compartment of bone marrow (known as bone marrow stromal cells, BMSCs) [4]. BMSCs’ osteogenic differentiation undergoes preosteoblasts, osteoblasts, mature osteoblasts, and ultimately the deposition and mineralization of the extracellular matrix. Trabecular bone is typically more metabolically active, given its greater surface area, and therefore is more sensitive to changes in bone remodeling balance [5]. Therefore, bones with more trabecular bone such as the vertebrae, femur and tibia, are more susceptible to osteoporosis [6].

So far, osteoporosis is mainly diagnosed depending on the complaints of back pain, radiographic changes of bone, and bone mineral density (BMD) at both the femur and lumbar spine [7]. However, radiographic alterations including the bone loss are usually signs of medium-late stage osteoporosis [8]. Therefore, biochemical markers involved in increased bone turnover have been proposed as potential indicators of the degree of severity of bone loss [9]. Accumulating data support markers that represent bone turnover to be correlated with osteoporosis progression. These markers are being studied as biomarkers for discovering bone loss, diagnosing early osteoporosis and monitoring disease progression [10].

In the current study, the secretion levels of several important cytokines were screened in peripheral blood serum of mice with age-related trabecular bone loss. Of these cytokines investigated, Galectin-1 (Gal-1) showed age-related decline in mice peripheral blood serum and bone marrow. Gal-1 is the first member of the β-galactoside-binding lectin family, galectins. Immunosuppressive function of Gal-1 has been confirmed in a number of in vivo and in vitro studies [11]. Targeted deletion of Gal-1 expression or function in tumor cells provokes immune response against the tumor and subsequent tumor rejection [12, 13]. Also, Gal-1 has been highlighted in growth and metastasis of solid tumors [14]. Accordingly, Gal-1 up-regulation in the tumor cells indicates poor prognosis of the disease [15]. An important role of Gal-1 in tumor angiogenesis has also been verified [16, 17]. Moreover, genetically engineered carcinoma-associated fibroblasts expressing low level of Gal-1 failed to support tumor progression [18]. High level of Gal-1 expression has been detected in BMSCs [19], which contributes to the T-cell regulating role of BMSC in vitro [20]. The functional role of Gal-1 in musculoskeletal system is a new research area with intense interests [21–23]. However, the role of Gal-1 in osteoporosis has not been investigated. It is possible that Gal-1 will play an important role in osteoporosis progression. Nevertheless, the current literature does not fully address the relationship between serum Gal-1 level and trabecular bone loss. Hence, in the current study, we sought to quantify serum Gal-1 level in young and aged mice to evaluate its utility as a biomarker for predicting age-related trabecular bone loss. In addition, the potential mechanisms underlying the age-related change in serum Gal-1 level in mice were elucidated preliminarily. More importantly, the clinical data of BMD and Gal-1 levels in peripheral blood serum were collected from male volunteers within various age groups and the relationship between them was investigated.

## Materials and methods

### Animals

On the basis of aged animal availability, male Balb/c and C57BL/6 mice (SIPPR-BK Laboratory Animal Co. Ltd., Shanghai, China) were housed under Specific-Pathogen-Free (SPF) conditions. All animal operations were approved by the Animal Ethics Committee of Shanghai Jiangong Hospital.

Fifteen animals were included in each group. Mice were euthanized using isoflurane inhalation anesthesia followed by cervical dislocation. The right femur and tibia and L1 vertebrae were prepared for micro computed tomography (micro-CT) scan; the left femur was used for BMSC, hematopoietic stem cell (HSC) and myeloid progenitor (MP) isolation. Peripheral blood serum was taken for the measurement of C-terminal telopeptides type I collagen (CTX-1) and propeptide of type I procollagen (P1NP) (Immunodiagnostic Systems plc, Tyne and Wear, UK).

### Micro-CT

BMD and bone microstructure of femur, tibia and L1 vertebrae were measured by Micro-CT (SkyScan 1172; Bruker-microCT, Kontich, Belgium) as previously described [24]. The bones were scanned at a low resolution, an energy level of 55 kVp, intensity of 145 μA, and a fixed threshold of 220. Trabecular bone volume fraction and microarchitecture of the distal femur, proximal tibia and L1 vertebrae were evaluated in the secondary spongiosa, starting proximately at 0.6 mm distal to the growth plate, and extending distally 1.5 mm. Approximately 230 consecutive slices were made at 10.5 μm interval at the distal end of the growth plate and extending in a proximal direction, and 100 contiguous slices were selected for analysis. Three-dimensional images were reconstructed by an in-house volume-rendering software, which renders 3D views of the CT scan from arbitrary view points and directions, as well as measures the parameters of bone microstructure. The main bone parameters are BV/TV (the relative volume of calcified tissue in the selected volume of interest (VOI), Tb. N (the number of trabecular bone) and Tb. Sp (trabecular bone separation; a measurement of the thickness of the spaces between the trabeculae).

### Antibody array

Absolute quantitative sandwich-based antibody arrays (RayBio® Mouse Cytokine Array Q-series) were employed to detect cytokines levels in mice peripheral blood serum as previously described [25]. The antibodies for detection use were biotin-labeled and combined to generate a single cocktail reagent for later use. The printed slides were placed in chamber assemblies so that each array could be incubated with a different sample. After incubation with a blocking buffer, the arrays were incubated with the peripheral blood serum samples. After washing to remove non-specific binding, the cocktail of biotinylated detection antibodies was added to treat the arrays. After washing, the array slides were incubated with a streptavidin-conjugated fluor (HiLyte Fluor™ 532 from Anaspec, Fremont, CA). The fluorescent signals were then visualized using a laser-based scanner system (GenePix 4200A, Molecular Dynamics, Sunnyvale, CA).

### Elisa

Gal-1 levels in bone marrow aspirates and peripheral blood serum of mice and peripheral blood serum of men were measured with ELISA kits (DY1245 and DY1152, R&D, Minneapolis, MN, USA) according to the manufacturer’s instructions.

### BMSC isolation and in vitro culture

BMSC was isolated as previously described [26]. The bone marrow from femur and tibia were suspended in cold PBS and passed through a 70 μm filter. The supernatants were kept to be used in ELISA. Filtered bone marrow cells were suspended in PBS with 2% FBS and 0.1 g/L phenol red and then enriched for lineage negative (Lin−) cells using the SpinSep system (Stem Cell Technologies, Vancouver, BC, Canada). The cells were incubated with a murine progenitor enrichment cocktail (Stem Cell Technologies) on ice for 30 min, washed, and then incubated with dense particles on ice for 20 min. The cells were then centrifuged at 1200 g for 10 min, and the cells at the density medium/PBS interface were collected.

Enriched BMSC were seeded onto culture plates at a density of 0.1 × 10^6^ cells/cm^2^ in α-MEM containing 100 units/ml penicillin (Gibco BRL, Rockville, MD, USA) and 100 μg/ml streptomycin (Gibco). The media were changed after 72 h and adherent cells were maintained in culture with twice weekly media changes.

### Immunofluorescence labeling and sorting of bone marrow cells

MACS LS columns (Miltenyi Biotec) were employed to enrich bone marrow by either positive isolation of c-Kit+ cells by labeling with anti-c-Kit magnetic beads or negative depletion of lineage-negative cells by labeling with biotin-conjugated antibodies specific for lineage markers followed by anti-biotin magnetic beads. Cells were collected from the enriched bone marrow for further sorting. Cells were gated for flow cytometry or cell sorting as follows: HSC, Lin^−^Sca-1^+^c-Kit^hi^IL-7Rα^−^Flt3^−^Thy-1.2^+^; MP, Lin^−^Sca-1^−^c-Kit^hi^IL-7Rα^−^.

### Western blot

Western blot was performed as previously described [27]. Cells were lysed on ice for 30 min in a buffer containing 50 mM Tris-HCl, pH 7.4, 150 mM NaCl, 1% Nonidet P^− 40^, and 0.1% SDS supplemented with protease inhibitors (10 g/ml leupeptin, 10 g/ml pepstatin A, and 10 g/ml aprotinin). The proteins were separated by SDS-PAGE, transferred to a PVDF membrane, and detected using anti-galectin-1 (#5418, Cell Signaling Technology, Danvers, MA, USA) and anti-GAPDH (#2118, CST). The proteins were visualized using an enhanced chemiluminescence system (GE Healthcare, Piscataway, NJ, USA). Densitometric analysis of immunoblots was performed using the ImageJ software provided by the National Institutes of Health (Developed by National Institutes of Health, Bethesda, Maryland, USA).

### Immunohistochemistry

As previously described [28], femur was fixed in 10% formalin, decalcified, and embedded in paraffin. Serial sections were cut every 5 μm. Slides were incubated with primary antibodies against mouse galectin-1 (#13888, CST) overnight at 4 °C. For immunohistochemical staining, a horse radish peroxidas-streptavidin detection system (Dako) was used to detect the immunoactivity followed by counterstaining with hematoxylin (Dako).

### Flow cytometry

As previously described [29], bone marrow cells of femur and tibia were harvested and pooled together. After red blood cells were lysed, the bone marrow cells were centrifuged and then the cell pellet was resuspended and fixed in 4% paraformaldehyde. Cells were permeabilized in 0.1% Triton X-100 and then blocked in blocking buffer (PBS with 3% FBS and 0.1% sodium azide) for 30 min on ice. Then the cells were incubated with anti-CD73 (12–0731-83, ThermoFisher scientific), anti-Sca1 (11–5981-85, ThermoFisher scientific) or isotype control for 1 h at 37 °C in dark room, and then washed twice with PBS with 0.1% BSA. Probes were analyzed using a BD Calibur flow cytometer and CellQuest software (Becton Dickinson).

### Study subjects

As previously described [30], the present study recruited and evaluated 92 Chinese men who had undergone routine physical check-up and DEXA in our hospital. Exclusion criteria were history of metabolic bone diseases such as chronic liver or renal failure, hyperthyroidism and rheumatoid arthritis; history of diseases affecting body weights or composition such as thyrotoxicosis, hypothyroidism; the presence of major debilitating disease; major cardiovascular events; none of the subjects had primary or secondary low levels of gonadal hormones or had treated with medicine capable of influencing BMD, weight and body composition such as thyroid hormones, glucocorticosteroids, bisphosphonates and anti-obesity drugs within the previous 3 months. In the end, 92 men were divided into three age groups: 30–39 (Group 1, *n* = 23), 45–54 (Group 2, *n* = 32), 65–74 (Group 3, *n* = 27) and > 80 (Group 4, *n* = 10) years of age.

The present study was conducted with the approval of the Ethics Committee of Shanghai Jiangong Hospital (Shanghai, People’s Republic of China). The investigators complied with all applicable regulatory and legal requirements and the Declaration of Helsinki from 1975 (as revised in 1983). Prior to inclusion in the study, each subject provided written informed consent and none of the subjects were involved in any study-related activity without giving appropriate written informed consent. Subject confidentiality was strictly maintained throughout the study.

### Evaluation of bone mineral density

As previously described [30], the BMD for all subjects was assessed with the help of Dual energy X-ray absorptiometry (DEXA) (Lunar iDXA, General Electric Company, Fairfield, CT, USA) scans at the femoral neck, total hip, and lumbar (L1-L4) spine. Bone area and bone mineral content scores were used to calculate BMD (g/cm^2^). All scans were acquired and analyzed by the same experienced operator, adhering to the guidelines provided by the manufacturer.

### Statistical analysis

Statistical significance was calculated by Student’s t-test for two-sample comparison. One-way ANOVA were used for multiple comparisons in software SPSS 16.0. Turkey test was used to find significant differences in ANOVA. Correlations of Gal-1 level in serum with trabecular bone volume fraction were calculated by Spearman correlation analyses. *p* < 0.05 were defined as significant. All data are presented as mean ± SD unless otherwise specified.

## Results

### Age-related trabecular bone loss in mice

The peak bone mass of mice is reached between 5 and 6 month of age [31]. Therefore, 6- and 18-month-old Balb/c and C57BL/6 mice were used to investigate age-related trabecular bone loss in the current study. Femur, tibia, and L1 vertebrae were collected. Micro-CT was employed to assess the trabecular bone microstructure.

**Fig. 1:**
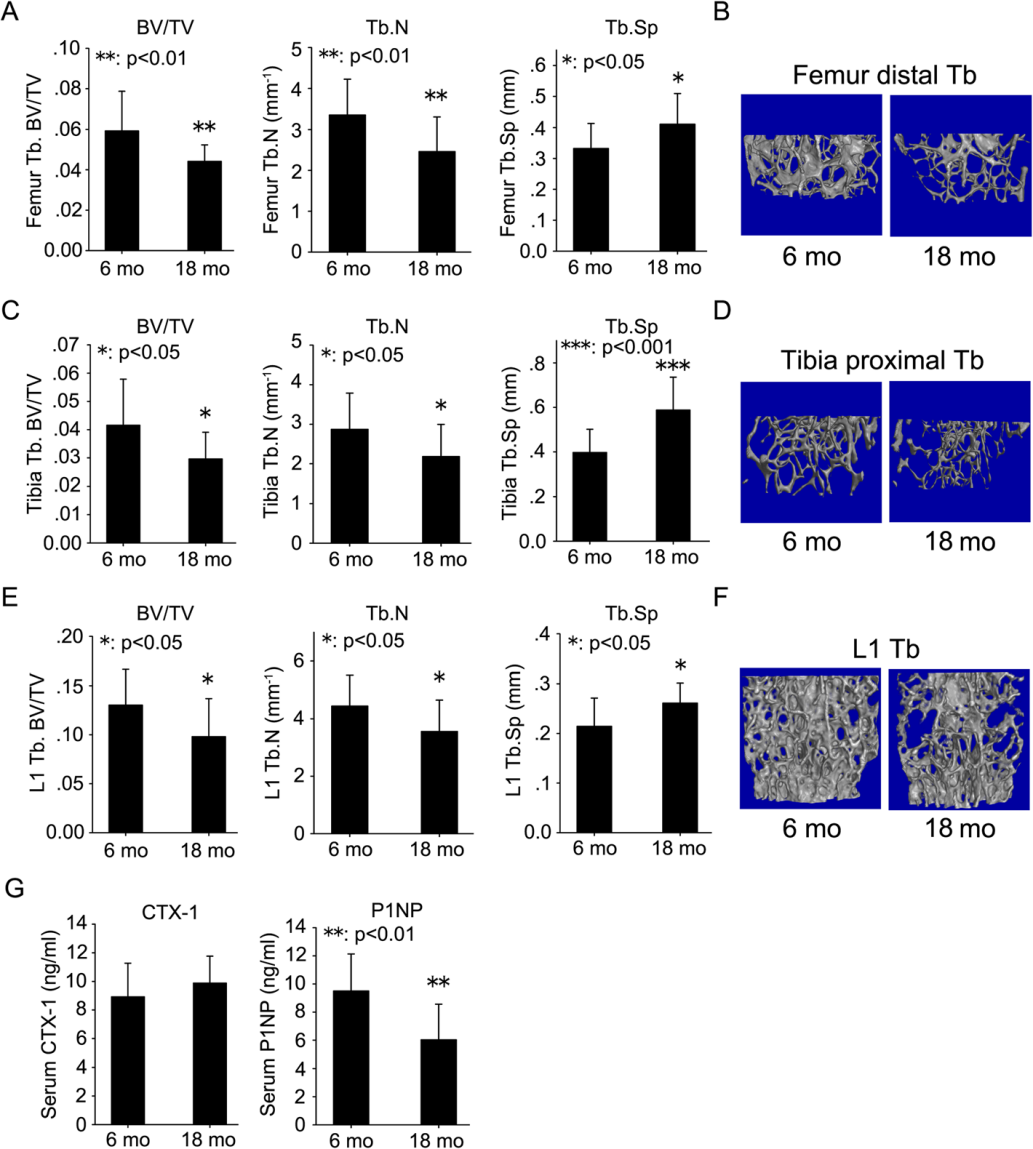
Age-related trabecular bone loss in mice. Femur (**a**), tibia (**c**) and L1 vertebrae (**e**) were harvested from 6- and 18-month-old mice. Micro-CT was employed to evaluate the trabecular bone microstructure. Trabecular bone parameters including BV/TV, Tb. N, and Tb. Sp were quantified according to micro-CT scans. Representative 3D reconstruction images of trabecular bone in the distal femur (**b**), proximal tibia (**d**), and L1 vertebrae (**f**) were displayed. Serum was prepared from peripheral blood of 6- and 18-month-old mice. ELISA was employed to quantify the levels of bone turnover markers including CTX-1 and P1NP (**g**). Data were shown as the means ± SD. *: *p* < 0.05, **: *p* < 0.01, ***: *p* < 0.001, 18 mo vs. 6 mo

In Balb/c mice, there was a 25% decrease in BV/TV, a 27% decrease in Tb. N and a 24% increase in Tb. Sp in the distal femur of 18-month-old mice compared with 6-month-old mice (Fig. 1a). There was a 29% decrease in BV/TV, a 24% decrease in Tb. N and a 48% increase in Tb. Sp in the proximal tibia of 18-month-old mice compared with 6-month-old mice (Fig. 1c). There was a 25% decrease in BV/TV, a 20% decrease in Tb. N and a 22% increase in Tb. Sp in the L1 vertebrae of 18-month-old mice compared with 6-month-old mice (Fig. 1e). 3-D reconstruction images of trabecular bone also showed the same pattern (Fig. 1b, d, and f).

In addition to micro-CT scan, bone formation and resorption markers in peripheral blood serum were analyzed by ELISA to determine the bone turnover status of 6- and 18-month-old mice. There was no significant change in CTX-1, a marker of bone resorption, between 6- and 18-month-old mice (Fig. 1g). However, a significant decrease (above 36%) in P1NP, a marker of bone formation, was observed in serum from 18-month-old mice compared with 6-month-old mice (Fig. 1g).

To eliminate the mouse strain bias, we investigated trabecular bone microstructure and bone turnover markers in serum in 6- and 18-month-old C57BL/6 mice in addition to Balb/c mice. The results from micro-CT and ELISA showed the comparable pattern with Balb/c mice, that is: there was significant trabecular bone loss in femur (Fig. S1A and S1B), tibia (Fig. S1C and S1D) and L1 vertebrae (Fig. S1E and S1F) and decrease in serum P1NP levels (Fig. S1G) of 18-month-old C57BL/6 mice compared with 6-month-old mice, although the change ranges between Balb/c and C57BL/6 mice were somewhat different.

### Correlation of age-related trabecular bone loss with decline in serum gal-1 level in mice

**Fig. 2:**
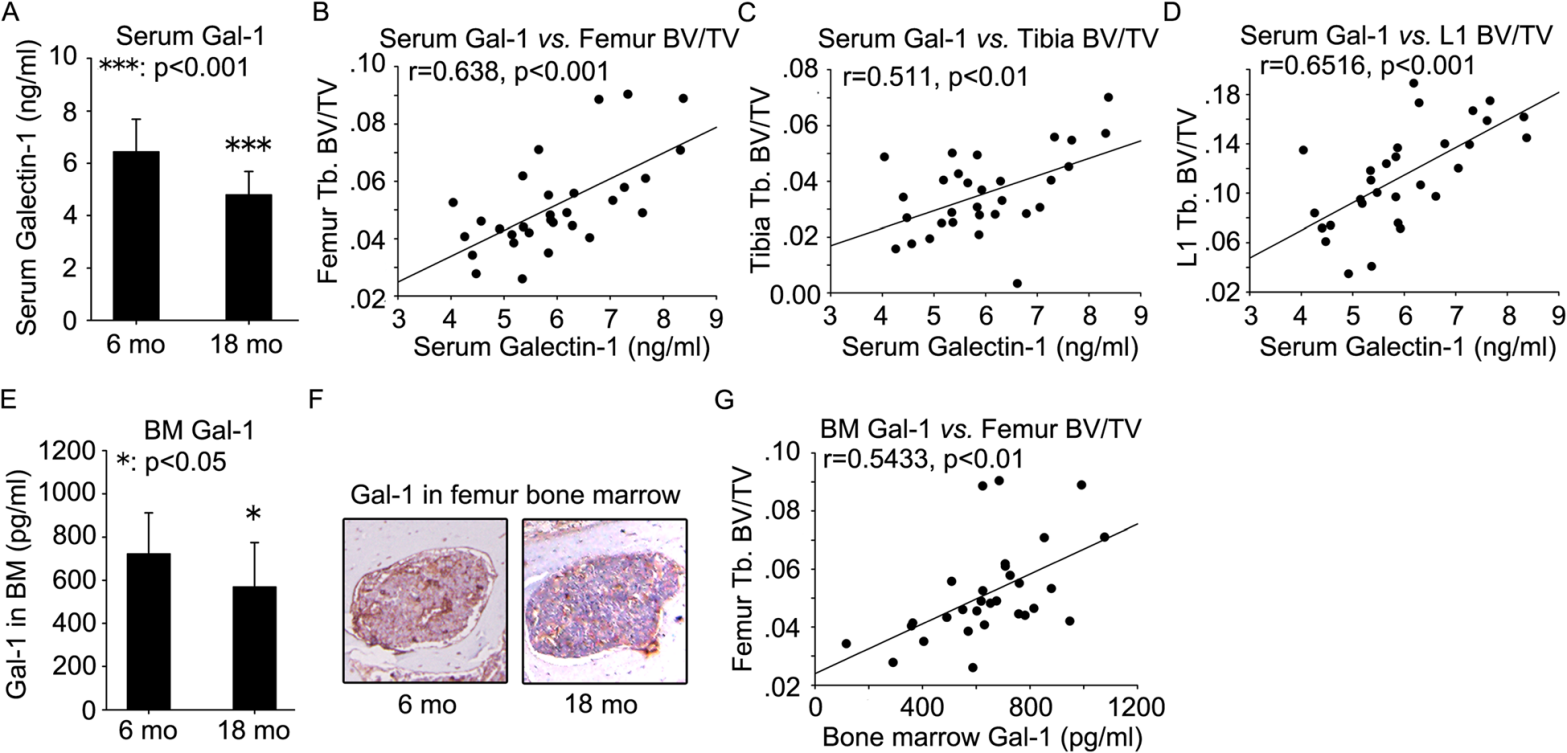
Age-related decline in Gal-1 levels in peripheral blood serum and bone marrow microenvironment in 18-month-old mice and the correlation of trabecular bone volume fraction with Gal-1 levels. Serum was prepared from peripheral blood of 6- and 18-month-old mice. ELISA was employed to quantify the levels of Gal-1 (**a**). The correlation of serum Gal-1 levels with BV/TV of femur (**b**), tibia (**c**), and L1 vertebrae (**d**) was analyzed. Femur bone marrow aspirates were prepared from 6- and 18-month-old mice. Gal-1 was quantified by ELISA (**e**). Immunohistochemistry assay was employed to investigate Gal-1 expression in femur bone marrow of 6- and 18-month-old mice (**f**). The correlation of bone marrow Gal-1 levels with BV/TV of femur (**g**) was analyzed. Data were shown as the means ± SD. *: *p* < 0.05, ***: *p* < 0.001, 18 mo vs. 6 mo

Serum was prepared from peripheral blood of 6 and 18-month-old Balb/c mice. Antibody array was employed to quantify the levels of several important cytokines. A significant decrease in Gal-1 secretion was observed in serum from 18-month-old mice compared with 6-month-old mice (Fig. 2a). There were no significant differences in secretion levels of TNFα (Fig. S2A), bFGF (Fig. S2B), IL-11 (Fig. S2C), IL-17 (Fig. S2D), CCL2 (Fig. S2E), CXCL1 (Fig. S2F) and SDF-1α (Fig. S2G) between 6- and 18-month-old mice. Likewise, age-related decline in serum Gal-1 level was also observed in 18-month-old C57BL/6 mice compared with 6-month-old mice (Fig. S3A).

Having observed age-related trabecular bone loss and decline in serum Gal-1 level in mice, we asked whether they were correlated. To answer this question, we explored the correlation of serum Gal-1 level with trabecular bone volume fraction (BV/TV) of femur, tibia and L1 vertebrae. The results showed that serum Gal-1 level was positively associated with femur BV/TV (*r* = 0.638, *p* < 0.001) (Fig. 2b and Table 1), tibia BV/TV (*r* = 0.511, *p* < 0.01) (Fig. 2c and Table 1) and L1 vertebrae BV/TV (*r* = 0.652, *p* < 0.001) (Fig. 2d and Table 1). In C57BL/6 mice, the results also showed that serum Gal-1 level was positively associated with femur BV/TV (*r* = 0.6814, *p* < 0.01) (Fig. S3B), tibia BV/TV (*r* = 0.6619, *p* < 0.01) (Fig. S3C) and L1 vertebrae BV/TV (*r* = 0.6312, *p* < 0.01) (Fig. S3D).

**Table 1:** Correlation of serum and bone marrow Gal-1 levels with trabecular bone BV/TV of femur, tibia and L1 vertebrae

Variables	Serum Gal-1 (ng/ml)		BM Gal-1 (pg/ml)	
	*r*	*p*	*r*	*p*
Femur BV/TV	0.638	< 0.001	0.543	< 0.01
Tibia BV/TV	0.511	< 0.01		
L1 vertebrae BV/TV	0.652	< 0.001		

*BM* bone marrow, *BV* bone volume, *TV* total volume

### Age-related decline in gal-1 level in bone marrow microenvironment in mice

Bone marrow aspirates were prepared from femur of 6 and 18-month-old Balb/c and C57BL/6 mice. ELISA was employed to quantify the Gal-1 level. A significant decrease in Gal-1 level was observed in femur bone marrow aspirates from 18-month-old mice compared with 6-month-old mice (Fig. 2e and S3E). The result from immunohistochemistry also showed that Gal-1 was down-regulated in femur bone marrow of 18-month-old mice compared with 6-month-old mice (Fig. 2f). We explored the correlation of Gal-1 level in bone marrow aspirates with femur BV/TV. The results showed that Gal-1 level in bone marrow aspirates was positively associated with femur BV/TV in Balb/c (Fig. 2g and Table 1) and C57BL/6 mice (Fig. S3F).

### Age-related decrease in BMSC number and down-regulation of gal-1 expression of BMSC

**Fig. 3:**
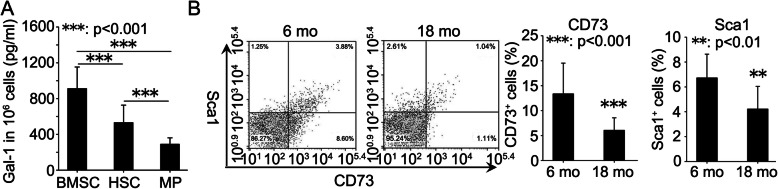
Age-related decrease in BMSC number in bone marrow microenvironment. Bone marrow stromal cell (BMSC), hematopoietic stem cell (HSC) and myeloid progenitor (MP) were harvested from femur bone marrow of 6-month-old mice and cultured in vitro. ELISA was performed to measure Gal-1 secretion in these supernatants (**a**). Flow cytometry was employed to measure the percentage of CD73 and Sca1 positive cells in femur bone marrow of 6- and 18-month-old mice (**b**). Data were shown as the means ± SD. **: *p* < 0.01, ***: *p* < 0.001

BMSC, HSC and MP within bone marrow were harvested from bone marrow of 6-month-old mice and cultured in vitro. ELISA was performed to measure Gal-1 secretion in these supernatants. BMSC secreted 1-fold higher Gal-1 as compared to cultured HSC and 2-fold higher Gal-1 relative to cultured MP (Fig. 3a). Flow cytometry was employed to measure the percentage of CD73 and Sca1 positive cells in bone marrow of 6- and 18-month-old mice. The results showed that the percentage of CD73 and Sca1 positive cells in bone marrow was significantly lower in 18-month-old mice compared with 6-month-old mice (Fig. 3b).

**Fig. 4:**
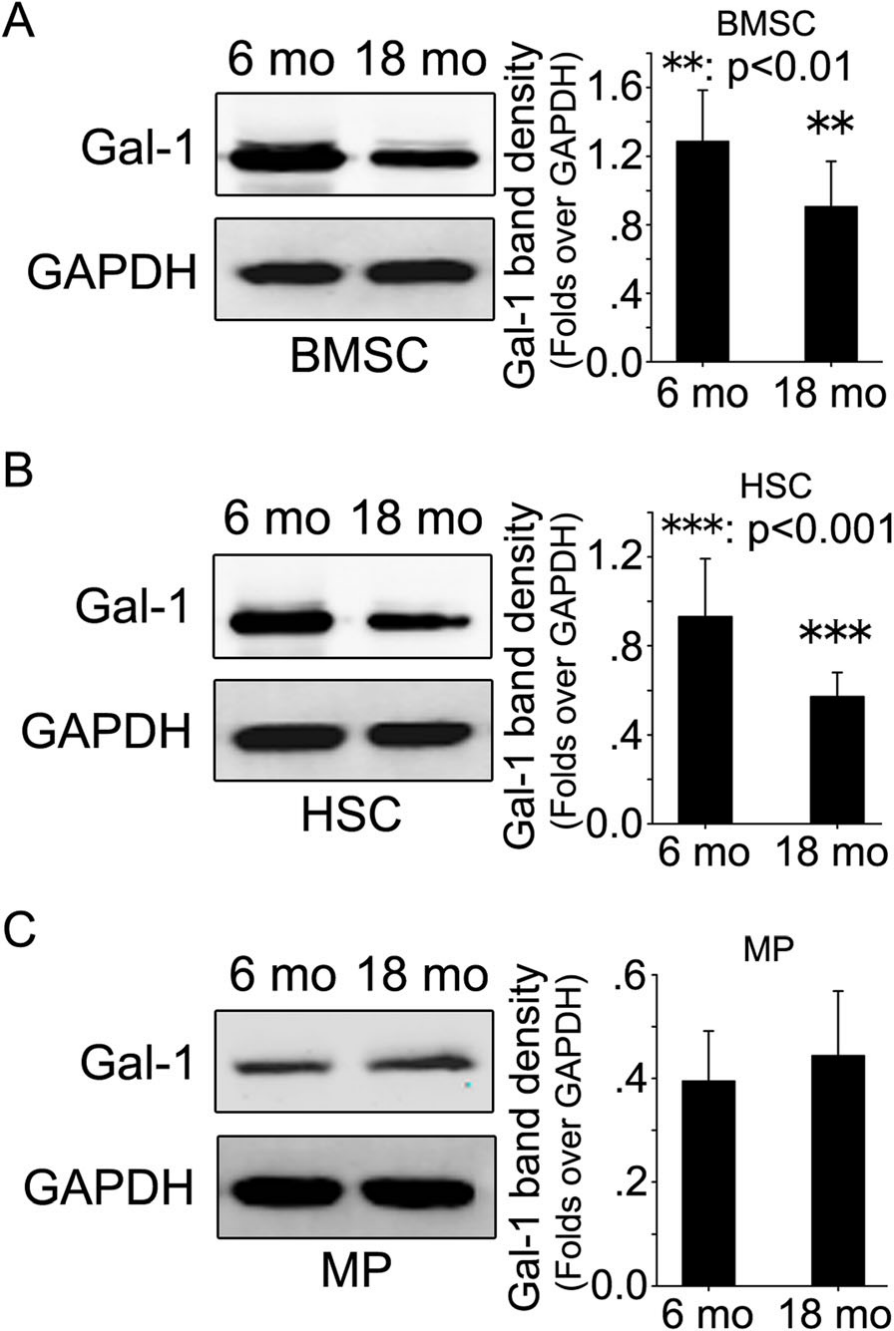
Comparison of Gal-1 protein expression of BMSC, HSC and MP in bone marrow between 6- and 18-month-old mice. Bone marrow stromal cell (BMSC), hematopoietic stem cell (HSC) and myeloid progenitor (MP) were harvested from femur bone marrow of 6- and 18-month-old mice. Gal-1 protein levels were investigated through western blot in BMSC (**a**), HSC (**b**) and MP (**c**) of femur bone marrow of 6- and 18-month-old mice. GAPDH were used as internal control. Original uncropped blots were presented in Fig. S5. Data are mean ± SD of three independent experiments. Densitometric analysis of immunoblots was performed using the ImageJ software

BMSC, HSC and MP were harvested from bone marrow of 6- and 18-month-old Balb/c and C57BL/6 mice. Western blot was employed to evaluate Gal-1 protein expression in these cell types. Significant down-regulation of Gal-1 protein expression was observed in BMSC (Fig. 4a and S4A) and HSC (Fig. 4b and S4B) from 18-month-old mice compared with 6-month-old mice. There was no significant difference in Gal-1 protein expression level in MP between 6- and 18-month-old mice (Fig. 4c and S4C).

### Correlation of BMD with serum Gal-1 level in men of different ages

**Fig. 5:**
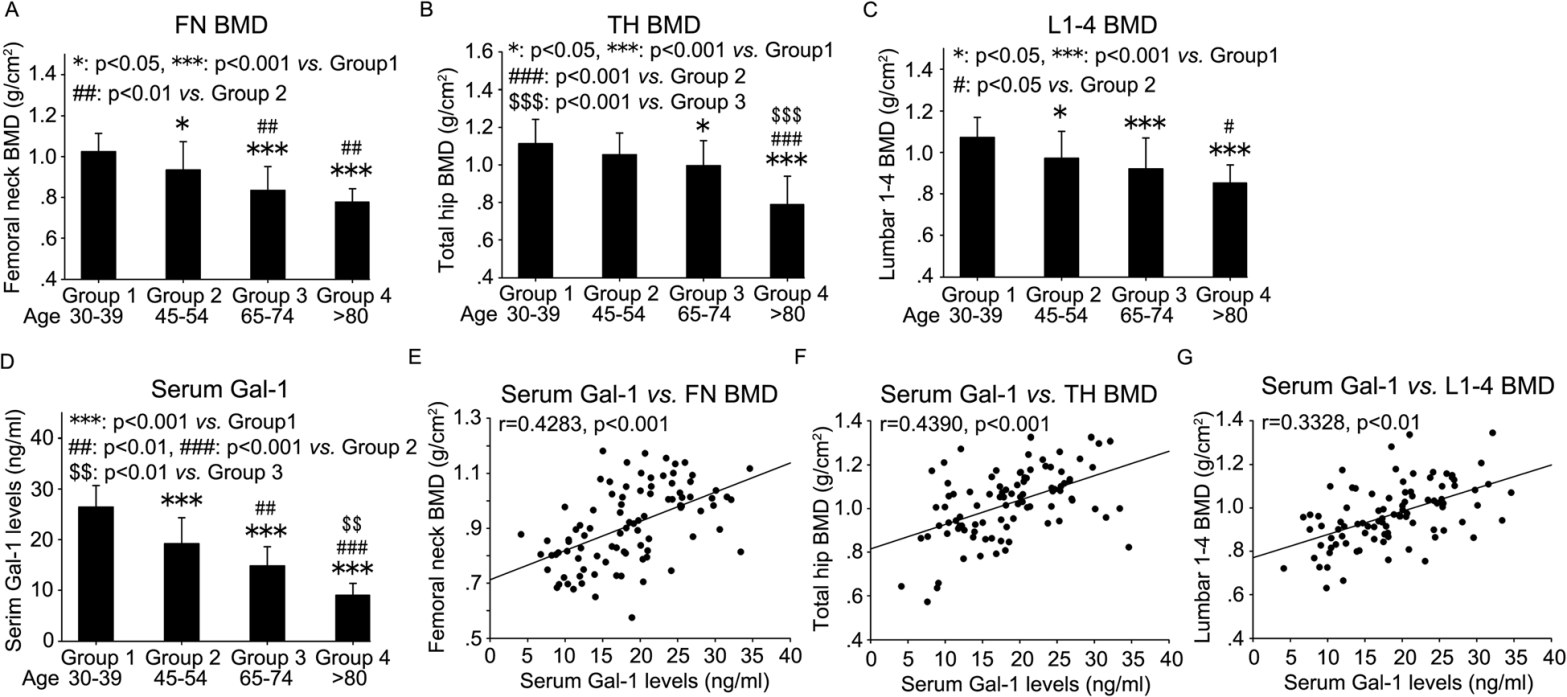
Age-related decline in BMD and Gal-1 levels in peripheral blood serum and the correlation between BMD and serum Gal-1 levels in men of different ages. DEXA was employed to measure BMD of femoral neck (**a**), total hip (**b**) and L1-L4 lumbar spine (**c**) of men within various age groups. ELISA was used to measure Gal-1 levels in peripheral blood serum from these men (**d**). The correlation of serum Gal-1 levels with BMD was analyzed (**e**, **f** and **g**). FN: femoral neck, TH: total hip, BMD: bone mineral density. Data were shown as the means ± SD. *: *p* < 0.05, **: *p* < 0.01, ***: *p* < 0.001

In the current study, a number of male volunteers of different ages were enrolled. The basic features of all the enrolled men were summarized in Table 2. Four age groups were divided: 30–39 (Group 1), 45–54 (Group 2), 65–74 (Group 3) and > 80 (Group 4) years old. There were no significant changes with regards to body mass index (BMI) among the four age groups (Table 2). DEXA was employed to measure BMD at the femoral neck (FN), total hip (TH) and L1-L4 lumbar spine. The results showed a gradual decline in FN BMD (Fig. 5a and Table 2), TH BMD (Fig. 5b and Table 2) and L1–4 BMD (Fig. 5c and Table 2) with age. ELISA was used to measure Gal-1 level in peripheral blood serum. Likewise, there was a gradual decrease in serum Gal-1 levels with age (Fig. 5d and Table 2).

**Table 2:** Comparison of demographic data, serum Gal-1 levels, and bone mineral density at the femoral neck, total hip, and lumbar spine among men within various age groups

	Group 1 (Age 30–39)	Group 2 (Age 45–54)	Group 3 (Age 65–74)	Group 4 (Age > 80)
Age (yrs)	35.4 ± 2.9	50.5 ± 2.3	68.7 ± 3.7	82.9 ± 2.0
BMI	24.36 ± 4.65	25.00 ± 4.10	24.13 ± 2.48	24.38 ± 2.00
FN BMD (g/cm^2^)	1.02 ± 0.089	0.93 ± 0.14*	0.83 ± 0.12***^/##^	0.78 ± 0.066***^/##^
TH BMD (g/cm^2^)	1.11 ± 0.13	1.05 ± 0.12	1.00 ± 0.13*	0.79 ± 0.15***^/###/$$$^
L1–4 BMD (g/cm^2^)	1.07 ± 0.096	0.97 ± 0.13*	0.92 ± 0.14***	0.85 ± 0.088***^/#^
Serum Gal-1 (ng/ml)	26.43 ± 4.25	19.18 ± 5.15***	14.80 ± 3.79***^/##^	9.00 ± 2.36***^/###/$$^

All data are conveyed as mean ± standard deviation. ^*^p < 0.05, ^***^p < 0.001, vs. Group 1; ^##^*p* < 0.01, ^###^*p* < 0.001, vs. Group 2; ^$$^*p* < 0.01, ^$$$^*p* < 0.001, vs. Group 3

*BMI* body mass index, *BMD* bone mineral density, *FN* femoral neck, *TH* total hip, *L1-4* L1-L4 lumbar spine

Having observed age-related BMD decrease and decline in serum Gal-1 level in men, we asked whether they were correlated. To answer this question, we explored the correlation of serum Gal-1 level with BMD of FN, TH and L1–4 lumbar spine. To varying degree, serum Gal-1 level was positively associated with FN BMD (*r* = 0.4283, *p* < 0.001) (Fig. 5e and Table 3), TH BMD (*r* = 0.4390, *p* < 0.001) (Fig. 5f and Table 3) and L1–4 BMD (*r* = 0.3328, *p* < 0.01) (Fig. 5g and Table 3).

**Table 3:** Correlation of serum Gal-1 levels with bone mineral density of femoral neck, total hip and L1-L4 in men

	Serum Gal-1 levels (ng/ml)	
	*r*	*p*
FN BMD (g/cm^2^)	0.4283	< 0.001
TH BMD (g/cm^2^)	0.4390	< 0.001
L1–4 BMD (g/cm^2^)	0.3328	< 0.01

*BMI* body mass index, *BMD* bone mineral density, *FN* femoral neck, *TH* total hip, *L1-4* L1-L4 lumbar spine

## Discussion

To the best of our knowledge, our findings are the first to illustrate that mice with age-related trabecular bone loss also have an age-related decline in serum Gal-1 level, demonstrating a correlation between this cytokine and bone loss. In addition, we found that Gal-1 level in bone marrow microenvironment also had an age-related decline, which is mainly resulted from an age-related decrease in BMSC number and attenuation of Gal-1 expression by BMSC in bone marrow microenvironment. These data suggest that Gal-1 is an important predictor of osteoporosis progression in aged mice.

In the past few years, some studies have been performed to elucidate the underlying mechanisms for both physiological and pathological bone turnover as well as in the field of “osteoimmunology” seeking to understand skeletal and immune system interactions further [32]. Bone and the immune system have been shown to have overlapping functions through shared receptors, soluble molecules, and signaling pathways [33]. Therefore, inflammation or the immune response may play an important role in osteoporosis.

Gal-1 is the first member discovered in Galectin family, and its expression is activated by varous physiological and pathological factors. Gal-1 inhibits monocytes and macrophages and migration of lymphocytes and neutrophils to inflammatory sites. In recent years, the role of Gal-1 in skeleton system has been being investigated, which mainly focused on Gal-1-mediated immunomodulation of BMSC and the role of Gal-1 in musculoskeletal system tumors. Andersen and colleague found [34] that Gal-1 was involved in alternative (M2-like) activation of macrophages in patients with multiple myeloma. In a previous study, the authors found that Gal-1 was expressed in 78% of osteosarcoma, 33% of chondrosarcoma, and 8% of the Ewing sarcoma family of tumors [35]. Gal-1 can modulate osteoblastic proliferation and differentiation. These effects were affected by IGF-I. Thus, Gal-1 is likely be involved in the osteoblastic response, caused by prostate cancer cells metastasizing into bone, by affecting the matrix mineralization [36]. Gal-1 regulated osteoclast activity with an increased resorption by Gal-1^−/−^ osteoclasts and decreased bone densities in Gal-1^−/−^ mice. An enhanced tumor development was observed in Gal-1^−/−^ mice compared to wild-type mice [37], suggesting that Gal-1 plays a functional role in stromal cells in myeloma microenvironment. However, whether Gal-1 is involved in the progression of osteoporosis remains unclear. To the best of our knowledge, our current study is the first to illustrate the relationship between Gal-1 and trabecular bone loss in osteoporosis.

In the current study, mice with age-related trabecular bone loss also had an age-related decline in serum Gal-1 level, demonstrating a correlation between this cytokine and trabecular bone loss. It is commonly recognized that trabecular bone loss in age-related osteoporosis is resulted from decreased bone formation, rather than increased bone resorption as in postmenopausal osteoporosis. Decreased bone formation may be due to a decline in the quantity or quality of osteoblasts, or both. For example, BMSCs from 24-month-old Balb/c mice showed decreases in proliferative ability [3]. Another study found that BMSCs from aged C57BL/6 mice have significant impairment in their osteogenic differentiation potential in addition to decreases in the proliferation ability [38]. Our results also supported these observations. In the current study the serum analysis revealed no significant change in bone resorption marker CTX-1 but a significant decrease in bone formation marker P1NP in 18-month-old mice compared with 6-month-old mice, suggesting that bone formation was reduced significantly in 18-month-old mice and bone resorption was almost not influenced by age.

In the current study, together with an age-related decline in Gal-1 level in mice peripheral blood serum, an age-related decline in Gal-1 level in bone marrow microenvironment was also observed. Moreover, this Gal-1 decline in bone marrow was associated with femur trabecular bone loss. In bone marrow microenvironment, BMSCs are responsible for giving rise to osteoblasts and hematopoietic-supporting stroma [39]. The differentiation potential of BMSCs is modulated via several secreted factors, immune molecules [1], and systemic hormones [40, 41] within the bone marrow niche. Moreover, BMSCs lie in the vicinity of HSC, whose microenvironment might be involved in the regulation of BMSCs differentiation and bone homeostasis. Several previous studies reported that Gal-1 is abundantly expressed in BMSCs as shown by transcriptomic and proteomic approaches [19, 42]. These reports support our data. In the current study, BMSCs, HSC and MP were isolated from mice bone marrow and cultured in vitro. The ability of Gal-1 secretion from these three cell types was evaluated. The results showed that BMSCs secreted 1-fold higher Gal-1 as compared to cultured HSC and 2-fold higher Gal-1 relative to cultured MP. Our data suggested that Gal-1 secreted from BMSCs constitutes the most important component of Gal-1 pool in mice bone marrow. Further, it is reasonable to believe that Gal-1 in bone marrow microenvironment constitutes the important component of Gal-1 pool in mice peripheral blood serum.

More importantly, age-related decrease in BMSCs number and attenuation of Galectin-1 expression of BMSCs were observed, which could explain the age-related decline in Gal-1 level in serum. It should be noted that whether BMSCs decrease in number with age remains controversial. For example, some studies have reported a decline in BMSCs number in elder individuals [43], whereas other scientists did not find any significant changes [7]. This could be due to different volumes of bone marrow aspirate used as well as different processing procedures [44, 45]. Moreover, age-related effects on BMSCs may be animal strain dependent. This is well known from studies performed on HSC, in which the repopulation ability of HSC is impaired with age in Balb/c but not in C57Bl/6 [46]. However, femur bone density showed a more significant decline with age in C57BL/6 than Balb/c mouse [47] suggesting that C57BL/6 mice might be more sensitive to age-related changes in bone phenotypes. This might explain why most of the studies to evaluate BMSCs number have been carried out in C57Bl/6 mice strain. Interestingly, the only study partly carried out using Balb/c mice showed a decrease in the number of CFU-F and CFU-O [48], which is supported by our current study utilizing Balb/c mice. In order to eliminate mouse strain bias, we also employed C57BL/6 mice in addition to Balb/c mice in the current study. We found that most of data obtained from Balb/c mouse could be reproduced in C57BL/6 mouse, including trabecular bone loss of femur, tibia and L1 vertebrae in aged mice, age-related decline in Gal-1 levels in serum and bone marrow in aged mice, positive association between Gal-1 levels and trabecular bone volume, and down-regulation of Gal-1 protein expression in BMSC and HSC from aged mice. It indicates that the data of current study is not mouse strain dependent. In the current study, we also found that the age-related trabecular bone loss in C57BL/6 mouse seems more severe than Balb/c mouse demonstrated by micro-CT. The previous study clearly show that phenotypically normal inbred strains of mice harbor remarkable differences in bone density, and in its components-mineral and volume [47]. These differences exist during the prime reproductive period for these stains and are not attributable to senescent changes. Since these genetically distinct strains of mice were raised in a controlled environment (diet, living space, light exposure, ambient temperature range, epizooites), the differences observed in bone parameters are primarily the result of genetic variation. Evidence for genetic variation in phenotypically normal bone density is readily available in the scientific literature. For example, among humans, in addition to twins and mother-daughter-granddaughter studies*,* racial group differences between blacks, whites and Asians are well known. In the mouse, population-based data on bone size and mineral content have been reported for some genetically homogeneous inbred strains. Murray et al. reported that C57BL/6 J mice had lower vertebral mass and lacked coupling of resorption and formation when compared with SENCAR strain mice [49].

One of the most interesting features of BMSCs from elder individuals appears to be their decreased osteogenic differentiation potential with increased bias toward adipogenic differentiation. The imbalance between osteogenic and adipogenic differentiation causes increased bone marrow adiposity and is evident in senile osteoporosis [50]. Therefore, the age-related decline in Gal-1 levels in bone marrow and peripheral blood serum present in our current study may be explained by at least two aspects of evidences: i) age-related decrease in BMSCs number; ii) age-related attenuation of Gal-1 expression by BMSCs and HSC.

The most important findings of current study come from the data of clinical samples. Eighty-two male volunteers within 30–74 years old were recruited and their BMD of femoral neck, total hip and L1-L4 lumbar spine and serum Gal-1 levels were measured. Positive correlation between BMD and serum Gal-1 levels was observed, which validated the data from mouse experiments. Many previous reports have demonstrated that there was a close relationship between circulating Gal-1 and many diseases. For example, increased Gal-1 serum levels have already been demonstrated in a number of systemic, non-central nervous system malignancies including head and neck squamous cell carcinoma [51], lung cancer [52], T cell lymphoma [53], classical Hodgkin lymphoma [54], and benign thyroid tumors [55]. In addition to various tumors, increased serum Gal-1 levels have been reported in rheumatoid arthritis (RA) patients [56]. Even serum Gal-1 levels are positively correlated with body fat in obese children [57]. To the best of our knowledge, we are the first to show positive correlation between serum Gal-1 levels and BMD in men and serum Gal-1 has great potential to be used to predict age-related bone loss.

Several previous studies have demonstrated the role of other members of galectin family, especially Gal-3, in skeleton system. For example, Nakajima K et al. [58] found that soluble Gal-3 in the bone microenvironment niche regulates bone remodeling through Notch signaling. Iacobini C et al. [59] revealed a wide range of age-dependent alterations in Gal-3^−/−^ mice including lower bone formation and higher bone resorption, accelerated age-dependent trabecular bone loss and reduced bone strength. Maupin KA et al. [60] identified Gal-3 as a negative regulator of bone formation. Vinik Y et al. [61] showed that mice overexpressing Gal-8 exhibit accelerated osteoclasts activity and bone turnover, which culminates in reduced bone mass, similar to cases of postmenopausal osteoporosis and cancerous osteolysis. These findings, together with our current work, provide a deeper insight into the role of galectin family members in skeleton biology.

Senile osteoporosis has become a worldwide bone disease with the aging of the world population. Unlike postmenopausal osteoporosis which is linked to menopause in women, senile osteoporosis is due to aging, hence, affecting both men and women. From the perspectives of bone remodeling, the pathological mechanisms differ between postmenopausal and senile osteoporosis. Postmenopausal osteoporosis is a high turnover disease, whereas senile osteoporosis is predominantly a low turnover disease [62]. As shown in the current study, age-related trabecular bone loss was associated with a decline in serum Gal-1 level. So what role dose Gal-1 play in postmenopausal osteoporosis, can Gal-1 be used as a biomarker in this case as well and even can Gal-1 be used to differentiate the difference between senile and postmenopausal osteoporosis? These questions are interesting, important and remain open, which need our further research.

## Conclusions

Age-related trabecular bone loss is associated with a decline in serum Gal-1 level in mice and men. The age-related decrease in BMSC number and attenuation of Gal-1 expression by BMSCs and HSC contributed to the decline in Gal-1 levels in bone marrow and serum of mice. The data from mouse experiments and clinical samples evaluation in the current study suggested Gal-1 had great potential to be a biomarker for discovering BMSCs senescence, diagnosing early osteoporosis and monitoring disease progression.

## Supplementary Information

**Additional file 1: Figure S1**. Age-related trabecular bone loss in C57BL/6 mice. **Figure S2**. Secretion of cytokines in peripheral serum of 6- and 18-month-old Balb/c mice. **Figure S3**. Age-related decline in Gal-1 levels in peripheral blood serum and bone marrow microenvironment in 18-month-old C57BL/6 mice and the correlation of trabecular bone volume fraction with Gal-1 levels in C57BL/6 mice. **Figure S4**. Comparison of Gal-1 protein expression of BMSC, HSC and MP in bone marrow between 6- and 18-month-old C57BL/6 mice. **Figure S5**. Uncropped blots of Fig. 4. Red lines indicate where they were cropped. **Figure S6**. Uncropped blots of Figure S4. Red lines indicate where they were cropped

## Data Availability

The data that support the findings of this study are openly available in Mendeley at 10.17632/68bdbzjytz.1.
