# Supplementary material for: Age-related trabecular bone loss is associated with a decline in serum Galectin-1 level
Source: BMC Musculoskelet Disord. 2021 Apr 27;22:394. doi: 10.1186/s12891-021-04272-y (PMC8080405; doi:10.1186/s12891-021-04272-y)
Supplement: Supplementary file 1 — Additional file 1: Figure S1. Age-related trabecular bone loss in C57BL/6 mice. Figure S2. Secretion of cytokines in peripheral serum of 6- and 18-month-old Balb/c mice. Figure S3. Age-related decline in Gal-1 levels in peripheral blood serum and bone marrow microenvironment in 18-month-old C57BL/6 mice and the correlation of trabecular bone volume fraction with Gal-1 levels in C57BL/6 mice. Figure S4. Comparison of Gal-1 protein expression of BMSC, HSC and MP in bone marrow between 6- and 18-month-old C57BL/6 mice. Figure S5. Uncropped blots of Fig. 4. Red lines indicate where they were cropped. Figure S6. Uncropped blots of Figure S4. Red lines indicate where they were cropped [file 12891_2021_4272_MOESM1_ESM.doc]

**Age-related trabecular bone loss is associated with a decline in serum Galectin-1 level**

Wenting Xu^1, 2^, Cheng Ni^2^, Yuxuan Wang^2^, Guoqing Zheng^2^, Jinshan Zhang^1^, Youjia Xu^1^, *

1 Department of Orthopaedics, The Second Affiliated Hospital of Soochow University, Suzhou, Jiangsu 215004, China

2 Department of Orthopaedics, Shanghai Jiangong Hospital, Shanghai 200083, China

*** Corresponding author:**

**Youjia Xu**

Tel: +86-0512-67783610, E-mail: xuyoujia@suda.edu.cn

**Running title:** Correlation of Galectin-1 and bone loss

**Conflict of interest statement**

Wenting Xu, Cheng Ni, Yuxuan Wang, Guoqing Zheng, Jinshan Zhang and Youjia Xu declare that they have no conflict of interests.


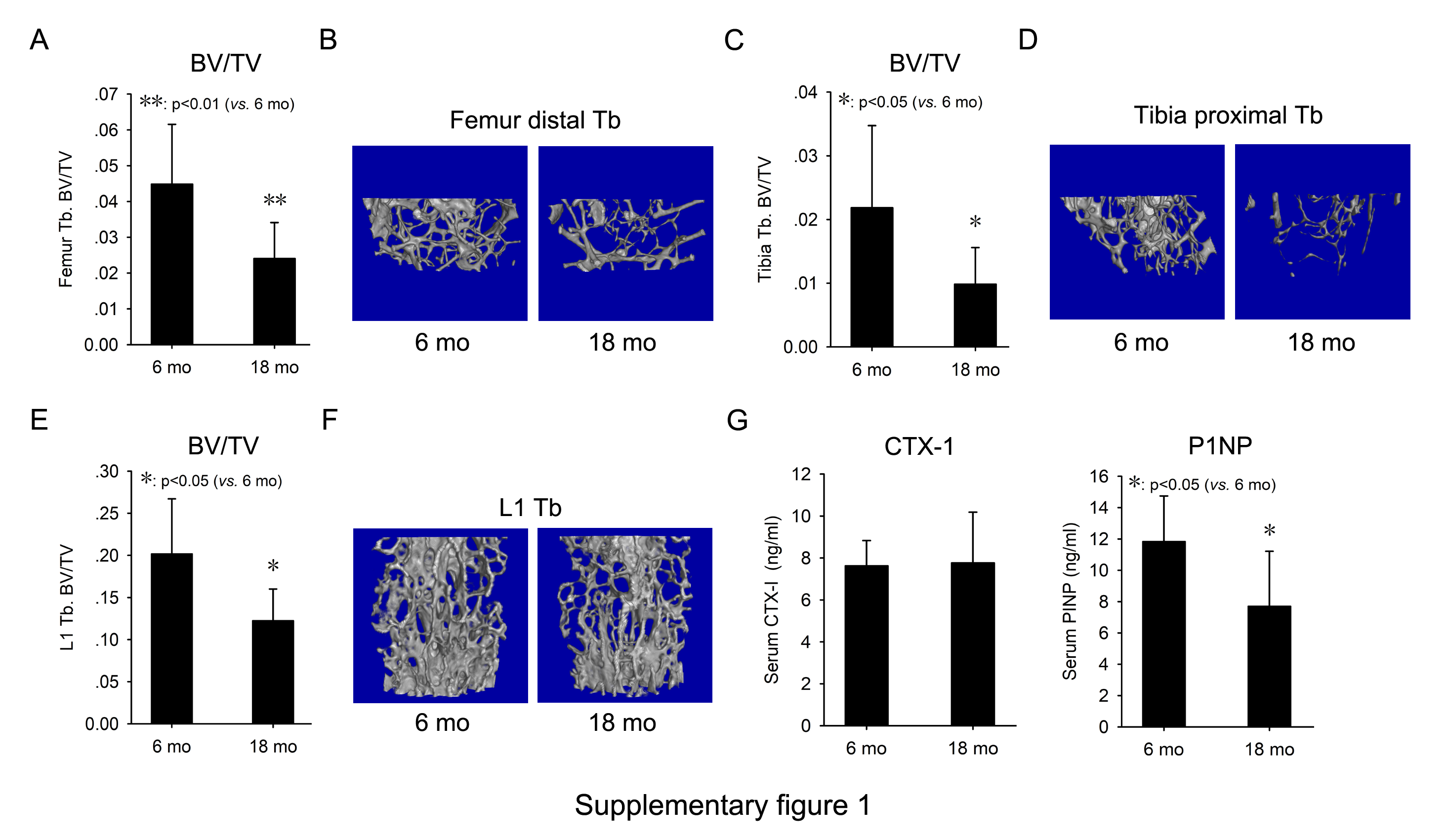


**Figure S1: Age-related trabecular bone loss in C57BL/6 mice.** Femur **(A)**, tibia **(C)** and L1 vertebrae **(E)** were harvested from 6- and 18-month-old C57BL/6 mice. Micro-CT was employed to evaluate the trabecular bone volume fraction (BV/TV). Representative 3D reconstruction images of trabecular bone in the distal femur **(B)**, proximal tibia **(D)**, and L1 vertebrae **(F)** were displayed. Serum was prepared from peripheral blood of 6- and 18-month-old C57BL/6 mice. ELISA was employed to quantify the levels of bone turnover markers including CTX-1 and P1NP **(G).** Data were shown as the means ± SD. *: p<0.05, **: p<0.01, 18 mo *vs.* 6 mo.


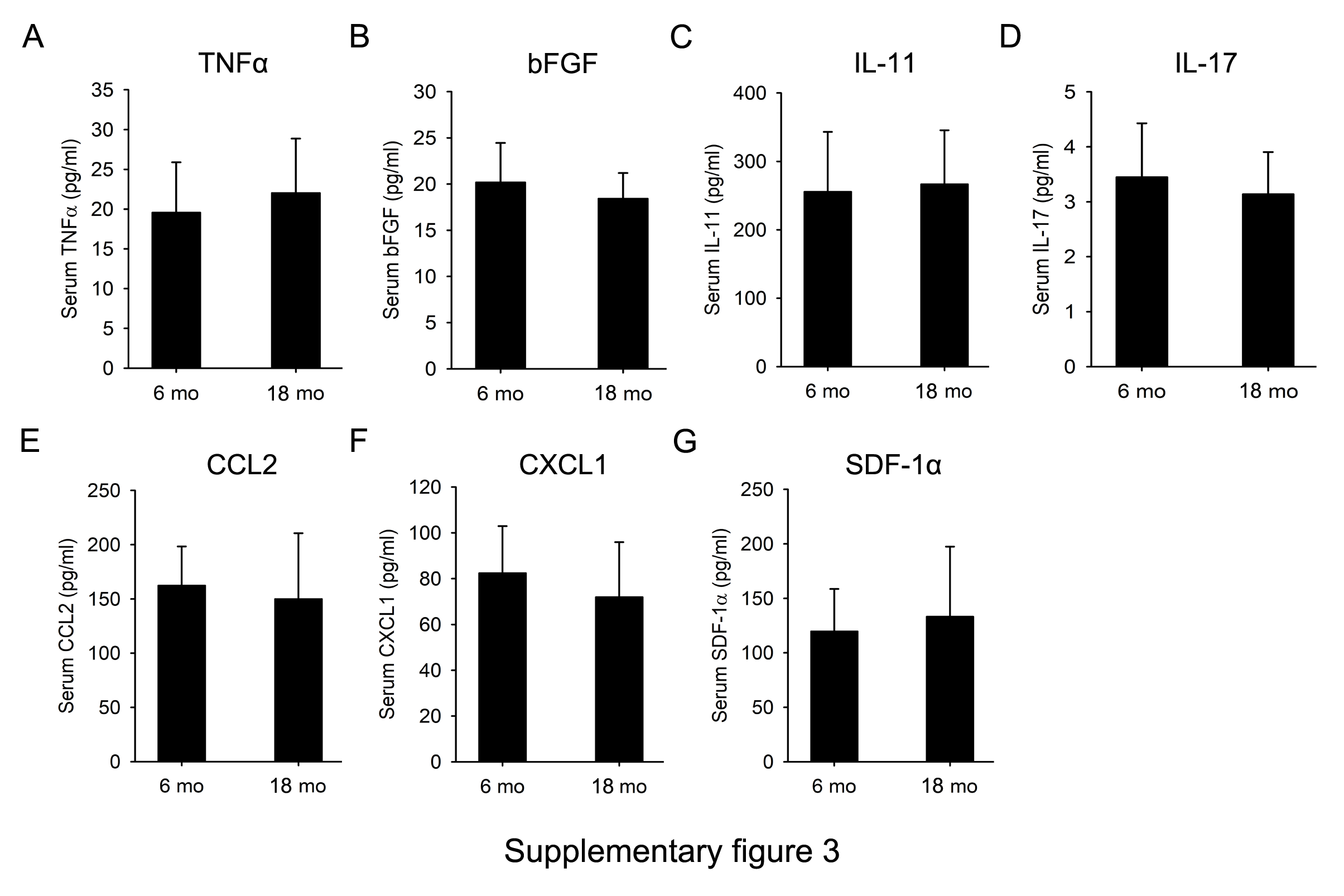


**Figure S2: Secretion of cytokines in peripheral serum of 6- and 18-month-old Balb/c mice.** Serum was prepared from peripheral blood of 6- and 18-month-old Balb/c mice. Antibody array was employed to quantify the levels of cytokines including TNFα **(A)**, bFGF **(B)**, IL-11 **(C)**, IL-17 **(D)**, CCL2 **(E)**, CXCL1 **(F)** and SDF-1α **(G)**. Data were shown as the means ± SD.


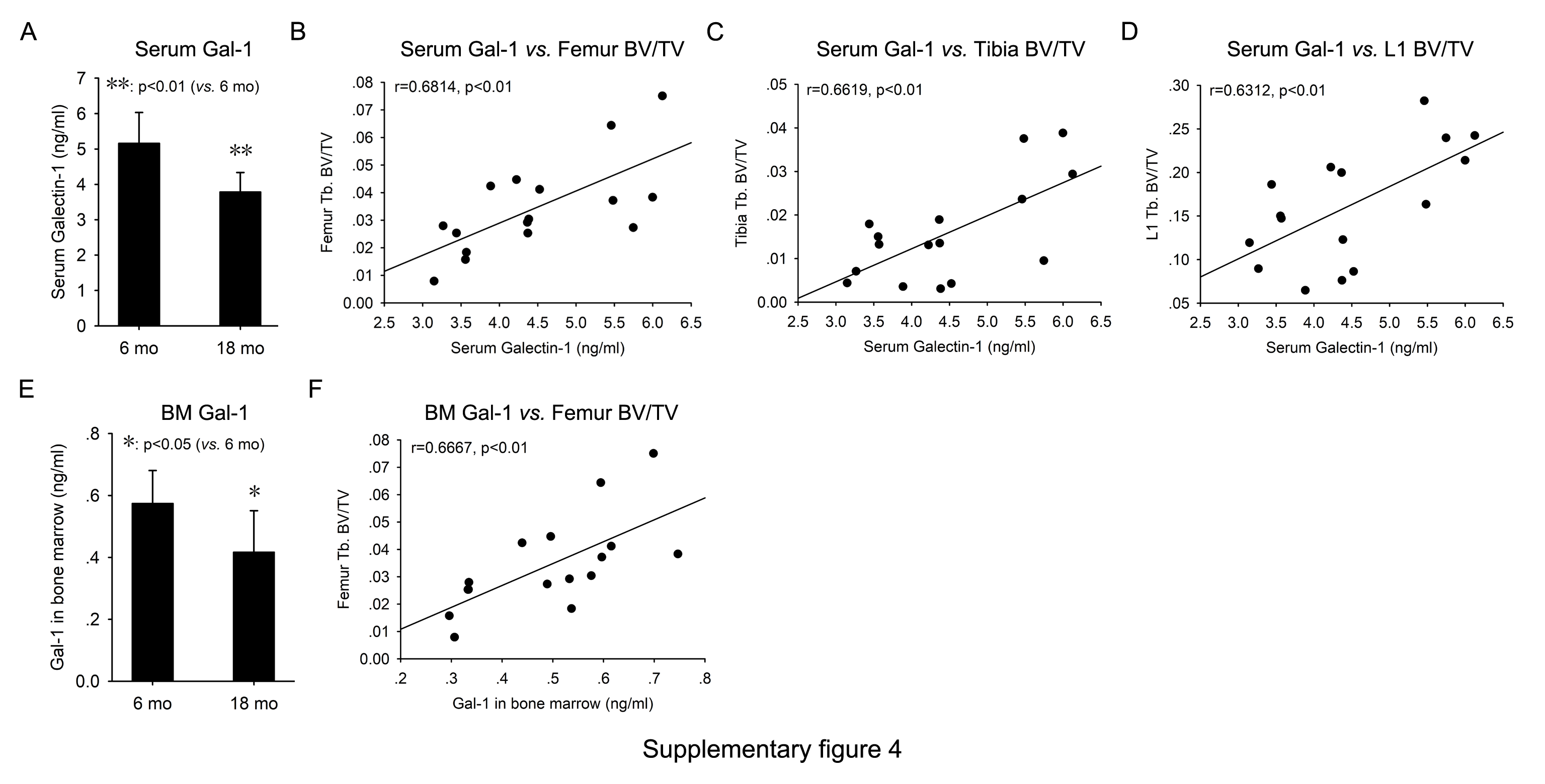
**Figure S3:** **Age-related decline in Gal-1 levels in peripheral blood serum and bone marrow microenvironment in 18-month-old C57BL/6 mice and the correlation of trabecular bone volume fraction with Gal-1 levels in C57BL/6 mice.** Serum was prepared from peripheral blood of 6- and 18-month-old C57BL/6 mice. Elisa was employed to quantify the levels of Gal-1 **(A).** The correlation of serum Gal-1 levels with BV/TV of femur **(B)**, tibia **(C)**, and L1 vertebrae **(D)** was analyzed. Femur bone marrow aspirates were prepared from 6- and 18-month-old C57BL/6 mice. Gal-1 was quantified by ELISA **(E)**. The correlation of bone marrow Gal-1 levels with BV/TV of femur **(F)** was analyzed. Data were shown as the means ± SD. *: p<0.05, **: p<0.01, 18 mo *vs.* 6 mo.


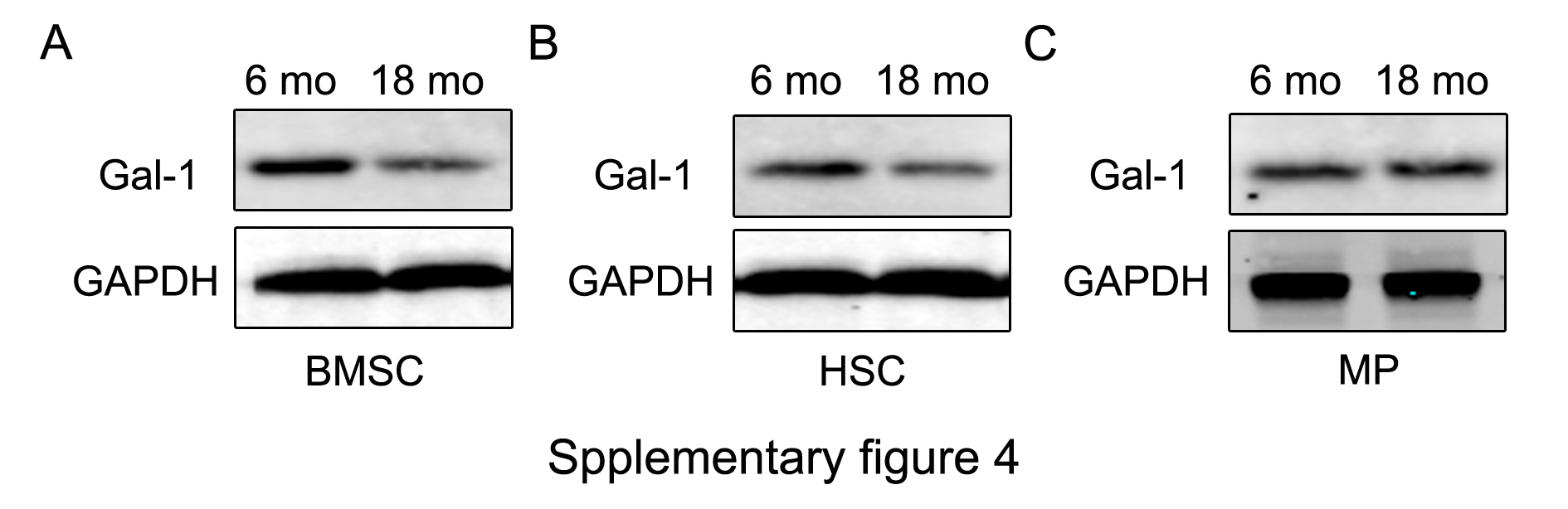


**Figure S4: Comparison of Gal-1 protein expression of BMSC, HSC and MP in bone marrow between 6- and 18-month-old C57BL/6 mice.** BMSC **(A),** HSC **(B)** and MP **(C)** were harvested from femur bone marrow of 6- and 18-month-old C57BL/6 mice. Gal-1 protein levels were investigated through western blot. GAPDH was used as internal control.


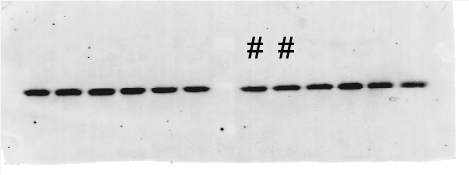

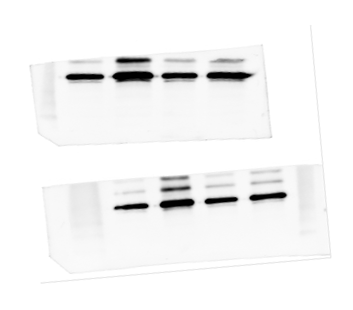


Figure 4A Gal-1 Figure 4A GAPDH


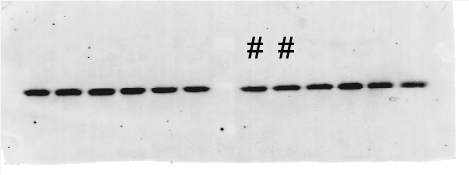

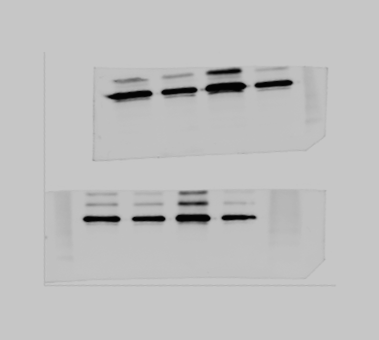


Figure 4B Gal-1 Figure 4B GAPDH


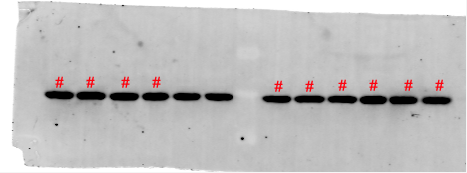


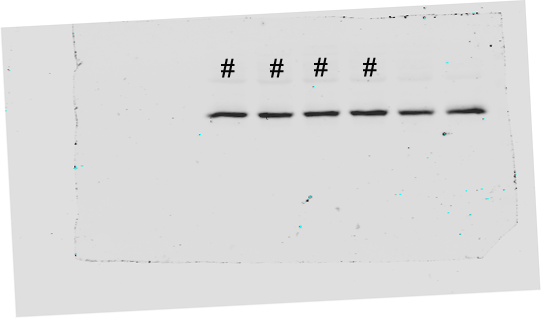


Figure 4C Gal-1 Figure 4C GAPDH

**Figure S5: Uncropped blots of Figure 4.** Red lines indicate where they were cropped.


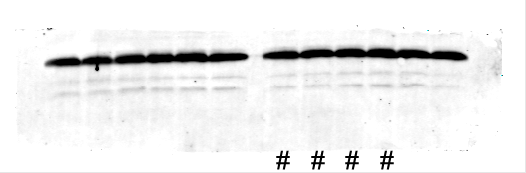

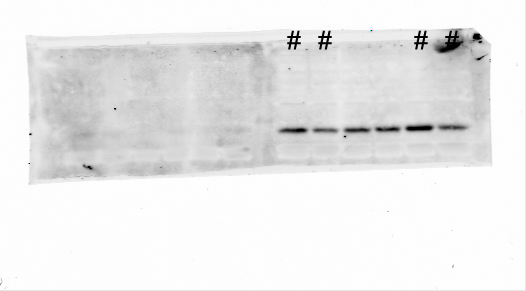


Figure S4A Gal-1 Figure S4A GAPDH


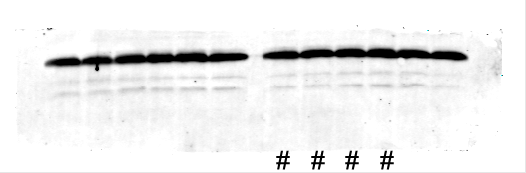

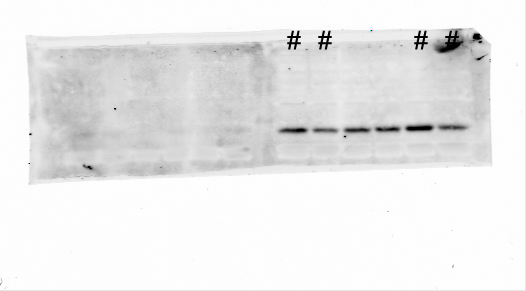


Figure S4B Gal-1 Figure S4B GAPDH


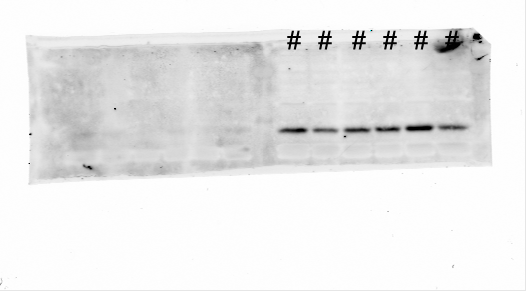

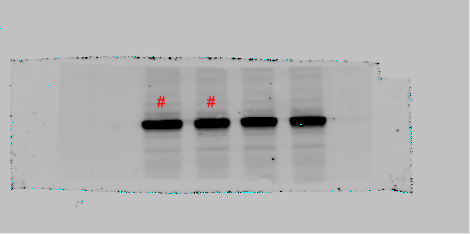


Figure S4C Gal-1 Figure S4C GAPDH

**Figure S6: Uncropped blots of Figure S4.** Red lines indicate where they were cropped.
